# Plasma metabolomic profiling of amino acids and polar lipids in Iranian obese adults

**DOI:** 10.1186/s12944-019-1037-0

**Published:** 2019-04-09

**Authors:** Minoo Bagheri, Abolghasem Djazayery, Farshad Farzadfar, Lu Qi, Mir Saeed Yekaninejad, Stella Aslibekyan, Maryam Chamari, Hossein Hassani, Berthold Koletzko, Olaf Uhl

**Affiliations:** 1 Department of Community Nutrition, School of Nutritional Sciences and Dietetics, Tehran University of Medical Sciences, No 44, Hojjat-dost Alley, Naderi St., Keshavarz Blvd, Tehran, 1416-643931 Iran; 2 Non-Communicable Diseases Research Center, Endocrinology and Metabolism Population Sciences Institute, Tehran University of Medical Sciences, Tehran, Iran; 3 Department of Nutrition, Harvard T.H. Chan School of Public Health, Boston, MA USA; 4 Department of Epidemiology and Biostatistics, School of Public Health, Tehran University of Medical Sciences, Tehran, Iran; 5 Department of Epidemiology, University of Alabama at Birmingham, Birmingham, AL USA; 6 Division of Metabolic and Nutritional Medicine, Ludwig-Maximilians-Universität München, Dr. von Hauner Children’s Hospital, 80337 Munich, Germany

**Keywords:** Amino acids, Metabolomics, Obesity, Polar lipids

## Abstract

**Background:**

Obesity, widely recognized as a serious health concern, is characterized by profoundly altered metabolism. However, the intermediate metabolites involved in this change remain largely unknown.

Objective: We conducted targeted metabolomics profiling to identify moieties associated with adult obesity.

**Methods:**

In this case-control study of Iranian adults, 200 obese patients were compared with 100 controls based on 104 metabolites profiled by a targeted metabolomic approach using liquid chromatography coupled to triple quadrupole mass spectrometry (LC-MS/MS). The analysis comprised acylcarnitines, diacyl-phosphatidylcholines (PCaa), acyl-alkyl-phosphatidylcholines (PCae), sphingomyelins (SM), lyso-phospholipids (LPC) and amino acids. We performed multivariable linear regression to identify metabolites associated with obesity, adjusting for age, sex, total energy intake, total physical activity, smoking, and alcohol consumption. The Bonferroni correction was used to adjust for multiple testing.

**Results:**

A pattern of 19 metabolites was significantly associated with obesity. Branched chain amino acids, alanine, glutamic acid, proline, tyrosine LPCa C16:1, PCaa C32:1, PCaa C32:2 and PCaa C38:3 were positively, while serine, asparagine, LPCa C18:1, LPCa C18:2, LPCe C18:0, PCae C34:3, PCae C38:4 and PCae C40:6 were negatively associated with obesity (all *p* < 0.00048).

**Conclusions:**

A metabolomic profile containing 9 amino acids and 10 polar lipids may serve as a potential biomarker of adult obesity. Further studies are warranted to replicate these findings as well as investigate potential changes in this profile after weight reduction.

## Introduction

Obesity, defined as body mass index (BMI) greater than 30 kg/m^2^, has increased from 28.8 to 36.9% among males and from 29.8 to 38% among females since 1980 worldwide [1]. This serious public health problem is associated with several metabolic disorders, including but not limited to type 2 diabetes, hypertension, dyslipidemia, and some cancers [2]. The burden of this epidemic is increasing especially dramatically in the Middle East, Southeast Asia, and Pacific Islands [2].

Much of the current etiologic understanding of obesity was derived from studies assessing the effects of genetic and environmental risk factors [3]. Although these studies have laid the groundwork at the population level, the biological mechanisms underlying obesity are not fully understood. Metabolomics, one of the systems biology methods used in biomarker discovery [4], strives to close this evidence gap by quantifying small molecules that may serve as products or substrates of enzymatic reactions involved in physiological processes.

Recent studies have used the metabolomic approach to generate preliminary evidence of specific metabolites that can distinguish lean and obese individuals. Specifically, plasma branched chain amino acids (BCAAs) were differentially present among obese and lean adults [4–6]. Furthermore, circulating acylcarnitines were shown to differ between obese and lean individuals and between diabetic and non-diabetic subjects [6]. The observed association was linked to a mitochondrial dysfunction, hindering effective transfer of cellular energy [6]. In addition to BCAAs and acylcarnitines, levels of phospholipids were also reported to be higher in obese compared to normal-weight individuals. This phenomenon was postulated to be due to decreasing β-oxidation of fatty acids leading to lipid accumulation [7].

To date, a limited number of metabolomics studies have focused on a simultaneous investigation of BCAAs, phospholipids, and acylcarnitines to establish a molecular signature of obesity. Of those, none were conducted in Middle Eastern populations, which have previously been shown to be especially vulnerable to the adverse sequelae of obesity [8]. Therefore, in this study, we sought to identify metabolite profiles associated with obesity using a more comprehensive, targeted metabolomics approach that quantified a large number of metabolites (104) in a case-control study of Iranian adults.

## Methods

### Study population

The study was approved by the Ethics Committee of Tehran University of Medical Sciences (IR.TUMS.REC.1394.792). The sample size was estimated to compare circulating isoleucine, a BCAA, that was well-characterized at the time of experimental design, between cases and controls. According to prior studies, the standard deviation (SD) of this metabolite was considered to be 15 μM [9]. The total number of required samples per group to detect a difference of 8 μM (with the power of at least 90% and the type one error of 5%) was estimated to be 75. Based on the population, the current study was not overlapping with any of the previously published papers.

Cases (*n* = 213, BMI ≥ 30 kg/m^2^) and controls (*n* = 100, (18.5 ≤ BMI < 25 kg/m^2^) were recruited either from Tehran Health Houses (basic health care providers) or by advertisements in local newspapers and websites and were matched based on sex and age (in annual increments). Individuals between 18 and 50 years of age who provided written informed consent, were included in the study. Data on age, sex, education, drug/supplements use, smoking, alcohol consumption, and medical history was gathered via questionnaire. Patients were not included if they 1) had lost more than five kg, followed a weight loss diet, or took weight loss medication during the 3 months prior to enrollment, 2) were pregnant, lactating or in menopause status at the time of recruitment, 3) used anti-hypertensive or lipid-lowering medications, 4) reported to have the following diseases: diabetes, cancers, as well as cardiovascular, renal, hepatic, respiratory, thyroid, autoimmune, and infectious diseases, 5) were heavy smokers (> 10 cigarettes/day) or alcohol drinkers (> = 20 g/day). Additionally, 12 obese adults with fasting blood glucose levels > = 126 mg/dL and one with plasma triglycerides = 885 mg/dL were excluded from the study, leaving the available sample size at 200 cases and 100 controls. After enrollment, trained staff instructed each participant on filling out three food records and asked them to present at the Diabetes Clinic of the Endocrinology and Metabolism Population Sciences Institute, Tehran University of Medical Sciences on a specified date.

### Compliance with ethical requirements

The study was approved by the Ethics Committee of Tehran University of Medical Sciences (IR.TUMS.REC.1394.792).

### Metabolomic measurements

Approximately three mL of fasting blood samples was collected from each study participant and added to an EDTA-containing tube. Plasma was separated after centrifugation (4 °C, 10 min, 2000 × g^3^) and stored at − 80 °C. Since there was not a qualified laboratory to simultaneously measure a large number of metabolites with a specified sensitivity in Iran, plasma samples were shipped in a three layer dry ice package (20 kg) to a metabolomics laboratory in Germany.

Liquid chromatography coupled to a triple quadrupole mass spectrometry (LC-MS/MS) [10, 11] was used to detect and quantify 505 metabolites: 22 amino acids, including 20 proteinogenic amino acids, ornithine, citrulline, acylcarnithines (acylcarnitineCx:y), hydroxylacylcarnitines, (acylcarnitineCx:y-OH), and dicarboxylacylcarnitines (acylcarnitineCx:y-DC), free carnitine, diacyl-phosphatidylcholines (PCaa), acyl-alkyl-phosphatidylcholines (PCae), sphingomyelins (SM), acyl-lyso-phospholipids (LPCa) and alkyl-lyso-phosphatidylcholines (LPCe), where Cx:y stands for lipid side chain, and x and y indicate the number of carbon atoms and double bonds, respectively, while OH and DC denote the hydroxyl group and two carboxyl groups, respectively.

First, 450 μL methanol including internal standards was added to 50 μL plasma to precipitate proteins. Polar lipids (PL), acyl-carnitines (acyl-Carn) and amino acids (AA) were analyzed using LC-MS/MS. To analyze PL, an aliquot of 30 μL of the supernatant was diluted by 500 μL methanol including 1,2-dimyristoyl-sn-glycero-3-phosphocholine and 1-tridecanoyl-2-hydroxy-sn-glycero-3-phosphocholine (Avanti Polar Lipids, Alabaster, USA) as internal standards. For this analyses, flow-injection with LC-MS/MS (1200 HPLC Agilent, Waldbronn, Germany coupled to 4000QTRAP Sciex, Darmstadt, Germany) was performed using 76% isopropanol, 19% methanol and 5% water as mobile phase. The position of double bonds and the arrangement of carbon atoms in the fatty acid side chains cannot be determined with this approach.

Acyl-carn analyses were performed by adding internal standards (D3-acetylcarnitine, D3-octanoylcarnitine and D3-palmitoylcarnitine (Cambrigde Isotope Laboratories, USA)) to 100 μL of the supernatant and injecting it to a LC-MS/MS system (1200 HPLC Agilent, Waldbronn, Germany coupled to 4000QTRAP Sciex, Darmstadt, Germany). Mass spectrometer equipped with an electrospray ionization source was used and the system was run in positive multiple reaction monitoring mode.

For the analyses of AA, amino acid standards set A, L-asparagine and L-tryptophane (Cambrigde Isotope Laboratories, Tewksbury, USA) were added to 50 μL of the supernatant. The amino acids derivatization was performed by adding 50 μL butanolic hydrochloric acid. After the solvent was evaporated, the residual was re-suspended in 100 μL water/ methanol/ formic acid (80:20:0.1). For the Analyses of AA butyl esters LC-MS/MS (1100 HPLC Agilent, Waldbronn, Germany coupled to API2000 Sciex, Darmstadt, Germany) was used.

Samples were assessed in six batches using six quality control samples in each batch. Mixed samples from the first batch were used as quality control pools, which were assessed among the study samples in each batch.

All metabolites were identified according to the Metabolomics Standards Initiative guidelines.

### Anthropometric and blood pressure measurements

A digital scale (Seca, UK) was used to measure the participants’ weight, recorded to the nearest 100 g. A wall-mounted tape (Seca, UK) was used to assess the patients’ height, reported to the nearest 0.5 cm. BMI was calculated using weight (in kilograms) over height (in meters) squared. Systolic and diastolic blood pressures were examined twice after 15 min of rest, using a sphygmomanometer fastened at patients’ right arms.

### Dietary intake and physical activity

Three day food records were completed during two consecutive weekdays and one weekend day by participants after appropriate instructions. A trained nutritionist checked the forms for accuracy with the help of the participants. Then, grams of food items were calculated and input into food analysis software (Nutritionist IV, N Squared Computing, San Bruno, CA, USA) to measure energy, macro-, and micro-nutrient intakes.

We used the short form of the International Physical Activity Questionnaire (IPAQ) to evaluate participants’ physical activity (PA) during the 7 days preceding the study visit. The Exceptional Talent Development Center of Iran University of Medical Sciences had translated the questionnaire into Farsi and confirmed its validity and reliability [12]. Median values and interquartile ranges for three forms of activities, including walking, moderate-intensity activities, and vigorous-intensity activities were reported and a combined total physical activity score was calculated, expressed in the metabolic equivalent of task (MET) value.

### Statistical analysis

As the intra-batch quality assessment, metabolites with coefficients of variations (CVs) over 25% or intraclass correlation coefficients (ICCs) less than 0.4 were excluded from the analysis. We also excluded metabolites for which more than 10% of samples did not have a valid measurement. Of 505 total measured metabolites, 401 were excluded based on these quality control procedures. For our inter-batch quality control analysis, we used SVA R package and Combat function [13] controlling for covariates (age, sex, total energy intake, total PA, smoking, and alcohol consumption). To achieve normality, metabolites which were retained after quality control were natural log-transformed.

To compare participants’ characteristics, we performed chi-square and Wilcoxon signed rank test for categorical and continuous variables, respectively. Multivariable linear regression was used to identify metabolites associated with obesity. These models, in which metabolites were considered as dependent variables, was adjusted for the covariates listed above. Pearson correlation was used to assess the correlations between obesity-associated metabolites. We implemented a Bonferroni correction to control for multiple testing, with the *p*-values cutoff defined as 0.05/104 metabolites = 0.00048. The statistical framework R 3.1.0 (www.r-project.org) was used to perform all the analyses.

## Results

### Population characteristics

A total of 200 obese patients and 100 normal weight adults with the mean (interquartile range (IQR)) age of 36 (31–42) participated in our study (Table 1). Cases and controls were statistically similar in age, sex, PA, and consumption of fat and protein. Compared to controls, obese patients had significantly higher BMI, as well as systolic and diastolic blood pressure (all *p* < 0.0001). Obese individuals were on average less educated (*p* < 0.0001), and consumed more total energy (*p* = 0.007) and carbohydrates (*p* < 0.0001) compared to normal weight individuals.

**Table 1:** Characteristics* of study participants

Charactristics*	Total (*n* = 300)	Control (*n* = 200)	Case (*n* = 100)	*p* ^†^
Gender				
Female [n(%)]	171 (57)	113 (56.5)	58 (58)	0.710
Age (y)	36 (31–42)	36 (31–42)	36 (31.8–42)	0.620
BMI (kg/m^2^)	32.4 (24.7–35.9)	23.1 (21.8–24.3)	34.1 (32.1–37.2)	<0.001
Education (y)	14 (12–16)	16 (12–18)	12 (12–16)	<0.001
DBP (mmHg)	80 (75–85)	71 (70–80)	80 (80–90)	<0.001
SBP (mmHg)	120 (110–125)	110 (100–118)	120 (115–130)	<0.001
Diet and Physical Activity				
Carbohydrate intake (g/day)	282.9 (227.6–348)	257.6 (207.3–307.2)	298.4 (235.7–362.4)	<0.001
Fat intake (g/day)	81.5 (62.7–102.7)	77.7 (60.2–96.4)	83.1 (63.1–104.4)	0.137
Protein intake (g/day)	72.2 (57.8–89.8)	72.2 (56.9–87.7)	72.495 (59–89.9)	0.472
Total caloric intake (kcal/d)	2128 (1773–2584)	2027 (1699–2306)	2197 (1807–2652)	0.007
Total physical activity (MET-hours/week)	422.2 (51.1–993)	495 (222.7–911.5)	396 (0–1070)	0.095

*Median(IQRs) or n (%)

†Comparison across cases (obese adults) and controls (lean adults). Wilcoxon signed rank test and Chi-square tests were used for continuous and categorical variables, respectively

Abbreviations: *BMI* body mass index, *SBP* systolic blood pressure, *DBP* diastolic blood pressure

### Relationships between metabolites and obesity

Associations of metabolites that passed quality control (*n* = 104) with obesity are shown in Table 2. In the multivariable analysis, a total of 19 metabolites were associated with obesity at the Bonferroni-corrected threshold. All of the significant associations were direct (i.e. case status was associated with the higher level of a particular metabolite), except for 8 metabolites, namely asparagine, serine, LPCa C18:1, LPCa C18:2, LPCe C18:0, PCae C34:3, PCae C38:4, and PCae C40:6.

**Table 2:** Associations* of plasma metabolites with obesity (*n* = 300)

Metabolite†	β	SE	*p*-Value^‡^	R^2^
Ala	0.174	0.033	3.66E-07	0.139
Asn	−0.080	0.023	4.57 E-04	0.074
Glu	0.266	0.025	6.96E-23	0.479
Ile	0.182	0.027	4.97E-11	0.379
Leu	0.119	0.022	1.63E-07	0.438
Pro	0.164	0.040	6.47E-05	0.130
Ser	−0.106	0.027	1.41 E-04	0.176
Tyr	0.161	0.026	1.44E-09	0.258
Val	0.134	0.021	4.82E-10	0.375
LPCa C16:1	0.154	0.038	8.21E-05	0.081
LPCa C18:1	−0.123	0.030	4.96E-05	0.098
LPCa C18:2	−0.217	0.034	8.51E-10	0.189
LPCe C18:0	−0.208	0.046	9.40E-06	0.124
PCaa C32:1	0.365	0.065	5.24E-08	0.178
PCaa C32:2	0.190	0.048	9.71E-05	0.157
PCaa C38:3	0.298	0.055	1.12E-07	0.197
PCae C34:3	−0.130	0.037	4.71 E-04	0.065
PCae C38:4	−0.125	0.035	3.65 E-04	0.088
PCae C40:6	−0.148	0.039	2.20 E-04	0.097

*Adjusted for age (years), sex, total calorie intake (Kcal/day), total physical activity (MET-minutes/week), smoking (cigarettes/day) and alcohol consumption (g/day)

†Metabolites were ln-transformed

‡ *p*-values were Bonferroni corrected at 0.05/104 metabolites = 4.8 × 10^−4^

Abbreviations: *SE* standard error, *Ala* alanine, *Asn* asparagine, *Glu* glutamic acid, *Ile* isoleucine, *Leu* leucine, *Pro* proline, *Ser* serine, *Tyr* tyrosine, *Val* valine, *LPCa* acyl-lysophosphatidylcholine, *LPCe* alkyllysophosphatidylcholine, *PCaa* diacyl-phosphatidylcholine, *PCae* acyl-alkyl-phosphatidylcholine

### Correlation matrix for the identified metabolites

**Fig. 1:**
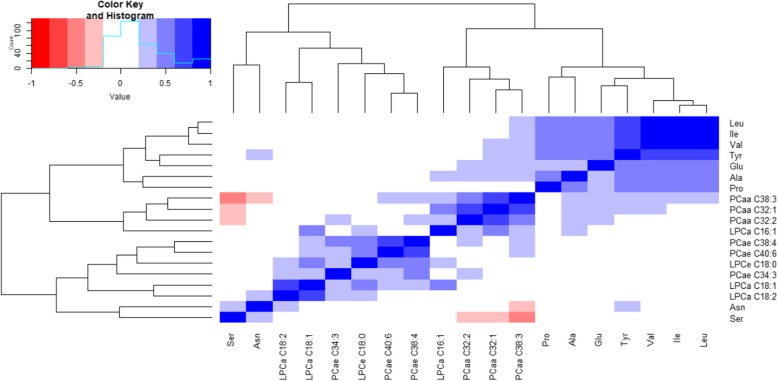
Correlation matrix for metabolites associated with obesity. Abbreviations: Ala: alanine, Asn: asparagine, Glu: glutamic acid, Ile: isoleucine, Leu: leucine, Pro: proline, Ser: serine, Tyr: tyrosine, Val: valine, LPCa: acyl-lysophosphatidylcholine, LPCe: alkyl-lysophosphatidylcholine, PCaa: diacyl-phosphatidylcholine, PCae: acyl-alkyl-phosphatidylcholine

Correlations within groups of the identified obesity-associated metabolites are presented in a matrix format (Fig. 1). The highest Pearson correlation coefficients were observed between isoleucine and valine (r = 0.87, *p* < 0.0001) and leucine and valine (r = 0.90, p < 0.0001). The lowest mean Pearson correlation coefficient was reported for serine (*r* = − 0.02). We had 323 independent correlation tests of which 195 were significant.

## Discussion

In this case-control study, we used a mass spectrometry-based metabolite profiling platform to identify a panel of 9 plasma amino acids and 10 plasma polar lipids associated with obesity in an adult Iranian population. We found that fasting plasma concentrations of BCAAs, alanine, glutamic acid, proline, tyrosine, diacyl-phosphatidylcholines, and LPCa C16:1 were higher in the obese participants, while circulating asparagine, serine, acyl-alkyl-phosphatidylcholines, and other lysophosphatidylcholines were higher in controls. On balance, we observed associations between obesity and both amino acids and polar lipids, consistently with findings from other populations.

Prior studies of metabolomic biomarkers in obesity have largely focused on a particular group of compounds rather than assessing a large number of metabolites at the same time [14–20]. However, emerging evidence suggests that not only metabolites’ absolute levels, but also metabolite relationships play a role in the biology of metabolism [21]. Therefore, it is crucial to simultaneously investigate a larger number of metabolites to arrive at a more accurate etiologic picture. Another important feature of metabolomics studies is the approach to measurements, with discrepant methods sometimes yielding inconsistent results. For example, using a different targeted metabolomic approach (electrospray ionization (ESI) tandem mass spectrometry) compared to ours (liquid chromatography coupled to triple quadrupole mass spectrometry(LC-MS/MS)), Oberbach et al. [22] reported a cluster of dissimilar amino acids and polar lipids compared to our findings.

**Fig. 2:**
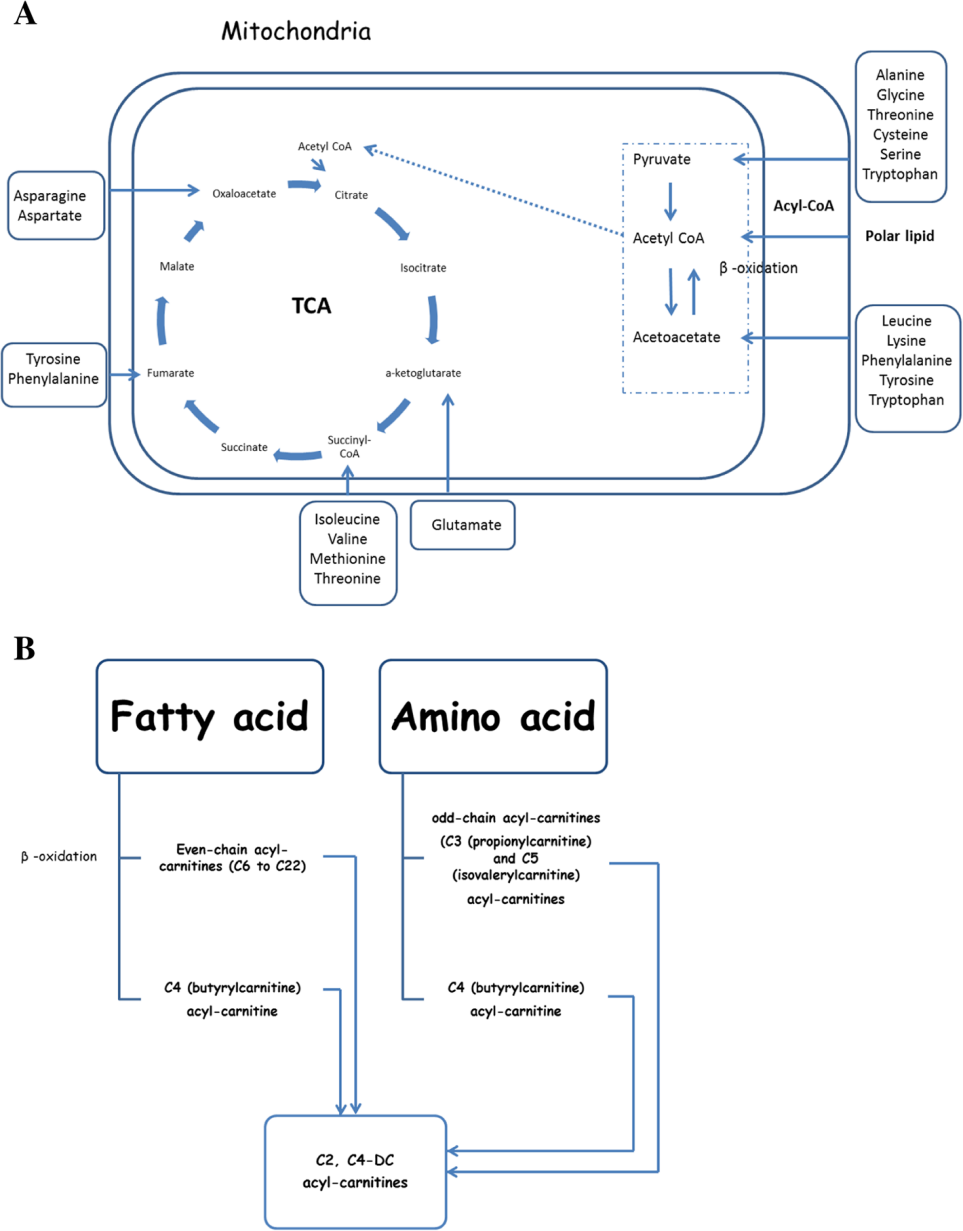
Schematic summary of amino acid and polar lipid catabolism in normal state (**a**) and during amino acid and polar lipid overload in obesity (**b**). Abbreviation: CoA: coenzyme A, TCA: tricarboxylic acid

**Fig. 3:**
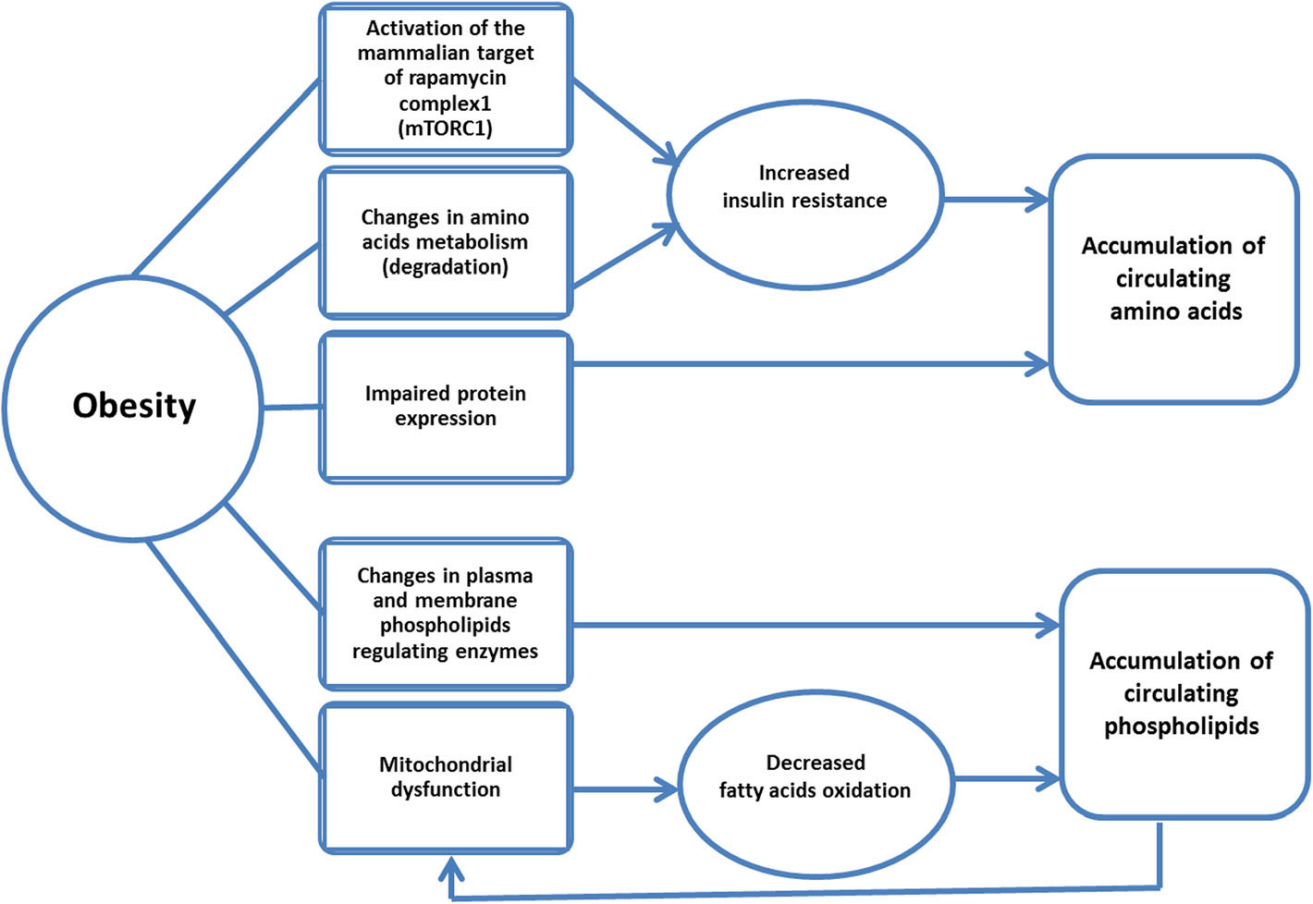
Selected metabolic derangements in obesity

Consistent with prior findings, we observed strong associations between BCAAs and obesity. Earlier, Jourdan et al. reported that levels of BCAAs were higher in individuals with higher BMI [23]. Moreover, BCAAs and aromatic amino acids have previously been proposed as biomarkers of metabolic syndrome [24]. Amino acids can be converted to one of seven metabolites: pyruvate, a-ketoglutarate, succinyl- Coenzyme A (CoA), fumarate, oxaloacetate, acetyl CoA, or acetoacetate. These metabolites are further degraded by the tricarboxylic acid cycle (TCA cycle) and oxidative phosphorylation [25] (Fig. 2a). The metabolites of amino acid (including BCAA) breakdown in a high catabolic rate are odd-chain acyl-carnitines such as C3 or propionylcarnitine and C5 or isovalerylcarnitine, while C4 or butyrylcarnitine is produced in both amino acid and fatty acid catabolism [26] (Fig. 2b). Apart from changes in amino acid degradation, elevated levels of BCAAs and other amino acids such as alanine, glutamic acids, proline, and tyrosine may be due to the activation of the mammalian target of rapamycin complex1 (mTORC1) or impaired protein expression [27–29]. Both protein degradation and activation of mTORC1 can lead to insulin resistance, which in turn can cause greater levels of circulating amino acids in obese patients [27, 28]. Also, the cellular transportation of BCAAs and the large neutral amino acids may be influenced by obesity-associated impairment in the expression of the LAT1 protein [29] (Fig. 3).

Our other findings related several polar lipids to obesity, also in line with previous research. For example, a recent study has shown an increase in BMI to be associated with a significant reduction in circulating lysophosphatidylcholines [30]. Polar lipids can be hydrolyzed to release fatty acids, which in turn are converted to acyl-carnitines [31]. The carnitine/acylcarnitine transporter is involved in the transportation of these compounds across the inner mitochondrial membrane, where fatty acids can be metabolized via β-oxidation [32] (Fig. 2a). Incomplete β-oxidation of fatty acids under mitochondrial fatty acid overload in obesity produces even-chain acyl-carnitines (C6 to C22) [26] (Fig. 2b). Previously published evidence indicates that under these conditions, mitochondrial redox status modulates metabolic pathways involved in fatty acid oxidation rather than amino acid catabolism, which leads to elevated levels of amino acids and their byproducts [33]. However, several mechanisms may explain the increased levels of polar lipids in obesity as well. First, increased oxidative stress followed by obesity-linked mitochondrial dysfunction may lead to decreased fatty acids oxidation, which may subsequently cause accumulation of circulating lipids in obesity [34]. This is likely to be a vicious cycle, leading to further mitochondrial dysfunction as this accumulation of circulating polar lipids is likely to further increase oxidative stress. [34] Second, obesity-related changes in enzymes regulating plasma and membrane phospholipids may also increase levels of circulating polar lipids [35, 36] (Fig. 3).

Regarding previous literature it is not clear if the identified metabolites are unique to Iranian population. However, there are gender and age-associated differences in metabolite profiles in various population groups [37, 38]. Therefore, beyond alterations in metabolomic approaches, any observed discrepancies in findings might be due to differences in the research population (in the study by Oberbach et al. [22], only men were included while in ours, we recruited both men and women). Furthermore, changes in diet and physical activity altered amino acid and phospholipid metabolisms [39–41]. However, according to our findings, there was not any significant association between metabolites and energy intake or physical activity after *Bonferroni* correction. Regarding diet composition, recent research revealed that changes in macronutrient intake can influence lipid and fatty acid chain in acylcarnitines [42]. But in our study although we measured acylcarnitines, we did not observe a significant difference, with respect to this group of lipid species, between cases and controls. Therefore, we concluded that the increased carbohydrate intake in the obese population might not contribute to the increased levels of any of the identified obesity-related metabolites.

Obese patients, compared to the control group, had significantly higher blood pressure. However, based on previous research [43], none of the identified obesity-linked metabolites has shown a significant association with blood pressure and therefore, might not contribute to higher blood pressure in the obese group.

Strengths of our research include a high-throughput profiling of metabolites that enabled evaluation of a large number of compounds, a state-of-the-art metabolomics technique (LC-MS/MS) with high sensitivity, rigorous correction for multiple testing, and a well-defined and unique study population at a high risk for cardiometabolic disease. However, our study also has some notable limitations. First, using a targeted approach may limit detection of obesity-associated metabolites. However, this method has been shown to have high sensitivity in previous research [44]. Second, the case-control, observational design precludes any inferences of causality rather than association. Future studies are warranted to replicate and expand our findings in both observational and experimental settings in diverse populations.

In conclusion, we have identified 9 circulating amino acids and 10 circulating polar lipids to be associated with obesity in an adult Iranian population. These findings merit further follow-up studies, e.g. as candidate metabolites for assessment of response to various therapeutic interventions [45]. Upon successful replication and validation, our findings may inform novel approaches to combating the metabolic sequelae of the obesity epidemic worldwide.

## Abbreviations

AA: Amino acid
acylcarnitineCx:y: Acylcarnithines
acylcarnitineCx:y-DC: Dicarboxylacylcarnitine
acylcarnitineCx:y-OH: Hydroxylacylcarnitine
Acyl-carnitines: Acyl-Carn
BCAA: Branched chain amino acid
BMI: Body mass index
CoA: Coenzyme A
CV: Coefficients of variation
ESI: Electrospray ionization
ICC: Intraclass correlation coefficient
IPAQ: International Physical Activity Questionnaire
IQR: Interquartile range
LC-MS/MS: Liquid chromatography coupled to triple quadrupole mass spectrometry
LPC: Lyso-phospholipid
LPCa: Acyl-lyso-phospholipid
LPCe: Alkyl-lyso-phosphatidylcholine
MET: Metabolic equivalent of task
mTORC1: mammalian target of rapamycin complex1
PA: Physical activity
PCaa: Diacyl-phosphatidylcholine
PCae: Acyl-alkyl-phosphatidylcholine
PL: Polar lipid
SM: Sphingomyelin
